# Characterization of Efficacy and Animal Safety across Four Caprine Disbudding Methodologies

**DOI:** 10.3390/ani11020430

**Published:** 2021-02-07

**Authors:** Kelly M. Still Brooks, Melissa N. Hempstead, Jessica L. Anderson, Rebecca L. Parsons, Mhairi A. Sutherland, Paul J. Plummer, Suzanne T. Millman

**Affiliations:** 1 Department of Veterinary Diagnostic and Production Animal Medicine, Iowa State University, Ames, IA 50011, USA; mnh3@iastate.edu (M.N.H.); jandersonvet2@gmail.com (J.L.A.); bparsons@iastate.edu (R.L.P.); pplummer@iastate.edu (P.J.P.); 2 Department of Clinical Sciences, Colorado State University, Fort Collins, CO 80523, USA; 3 AgResearch Ltd., Ruakura Research Centre, Hamilton 3214, New Zealand; kiwi.inus@gmail.com; 4 Department of Veterinary Microbiology and Preventive Medicine, Iowa State University, Ames, IA 50011, USA; 5 Department of Biomedical Sciences, Iowa State University, Ames, IA 50011, USA

**Keywords:** goat, disbudding, heat cautery, caustic paste, clove oil, cryosurgical

## Abstract

**Simple Summary:**

Disbudding of dairy goat kids is a routine, necessary, but painful husbandry procedure. Heat cautery disbudding is the industry standard since it is effective and can be rapidly performed by lay personnel on large numbers of kids. Efforts to improve welfare associated with heat cautery disbudding commonly focus on adjunct anesthesia and analgesia but there are often technical, legal, and safety barriers to routine use of these adjunct therapies in production settings. This project explores four alternative methods to heat cautery disbudding for safety, efficacy, and vocal evidence of duress during the procedure. We found that heat cautery was the most effective, was similar to the sham procedure for vocalization count during the procedure, and did not cause any serious or lasting complications. Clove oil injection, short-term topical application of caustic paste, and two cryosurgical methods were not consistently effective; additionally, the latter two created significantly more vocalization efforts. Clove oil injection was associated with several unexpected and severe complications including unintended tissue necrosis, temporary paresis, skull defects, meningitis, and death. Collectively, we did not find that any of the alternative methods of disbudding provided a feasible option over heat cautery to improve welfare associated with the disbudding process.

**Abstract:**

There is a strong industry demand for technically simple and highly efficacious alternatives to heat cautery disbudding in goat kids that can be performed as a stand-alone procedure without adjunct anesthesia, and that result in improved overall welfare through reduced acute pain, reduced tissues healing interval, and a consistent safety record. The objective of this study was to consider the net effect of disbudding techniques on goat welfare by examining vocalization frequency, long-term efficacy and animal safety associated with four alternative caprine disbudding methods against sham-disbudded and heat-cautery controls. Sixty-five commercial male dairy kids were disbudded at 3–10 days of age with one of six disbudding treatments (clove oil injection, caustic paste, two cryosurgical methods, heat-cautery, and sham procedure). Heat cautery was 91% effective, caustic paste was 55% effective, and the other treatments were ineffective. Heat cautery and sham procedures resulted in similar vocalization efforts; freezing with a liquid-nitrogen cooled iron resulted in significantly greater vocalization numbers. No unintended paste transfer injuries were observed with short-term application of the caustic paste. Heat cautery resulted in numerous superficial infections but no permanent injury. Clove oil injection was associated with several unexpected and severe complications including unintended tissue necrosis, temporary paresis, skull defects, meningitis, and death. Collectively, we did not find that any of the alternative methods of disbudding provided a feasible option over heat cautery to improve welfare.

## 1. Introduction

Caprine disbudding is commonly performed for safety considerations or as a requirement by show organizations. Because the intersex condition is associated with breeding for selection of polled goats, the majority of dairy goats are born horned and are disbudded by producers during the first one to two weeks of life, most often without adjunct anesthesia or pain relief due to technical, safety, and legal barriers in the United States. Heat cautery disbudding is the industry standard since it is effective and can be rapidly performed by lay personnel on large numbers of kids; it is typically viewed as a “brief grief” [1]. Still, the procedure is painful as evidenced by both an increase in cortisol and pain behaviors after disbudding [2,3,4]. Furthermore, there is risk of thermal injury to the brain [5,6], the healing lesions are susceptible to infection [7], and remain more sensitive to nociceptive pressure until re-epithelialization, which can take 5–9 weeks to occur [8]. Although adjunct anesthesia and analgesia can reduce acute pain associated with hot iron disbudding [9,10,11], chronic pain is more challenging to address in production settings and it is important to look beyond acute pain when assessing welfare. Procedural complications and other animal safety considerations may increase or prolong pain, or create additional suffering through illness or death. Similarly, poor efficacy may result in secondary dehorning procedures, ongoing scur management challenges, or exacerbate social conflict within the herd. There is a strong industry demand for technically simple and highly efficacious alternatives to heat cautery disbudding that can be performed as a stand-alone procedure without adjunct anesthesia, and that result in improved overall welfare through reduced acute pain, reduced tissues healing interval, and a consistent safety record.

Three alternative options exist that may either induce less pain and distress than heat cautery disbudding or improve cumulative welfare by reducing the interval to wound healing or risk of iatrogenic injury. The first potential alternative, caustic disbudding paste, has been marketed as less painful than hot-iron disbudding. However, in several studies goats demonstrated greater acute pain sensitivity, cortisol, and expression of pain behaviors with caustic paste as compared to hot iron disbudding [3,4,12]. An additional consideration in group housed or dam-raised goat operations is the risk of cohort injury due to unintended transfer of the caustic paste. One resolution to this problem is the application of local barrier bandages. Short-term paste application followed by paste removal has the potential to reduce the opportunity for iatrogenic transfer and injury or to reduce the duration of pain from the chemical burn. However, little information is available about the optimal duration of caustic paste or if a shortened period could incur the risk of a failure to disbud. If effective, this modified technique may provide a compromise of improved welfare and efficacy in goat kids.

Freeze disbudding has been successfully performed in crossbred *Bos indicus* calves [13] though replications in *Bos taurus* dairy breeds have been less successful [14,15]. While pain responses to cryosurgical procedure have not been well characterized in the literature for goats, in cattle, the freeze branding procedures is associated with reduced escape-avoidance reactions and physiologic indicators [16,17] and less acute pain, discomfort, and tissue inflammation [18,19,20] than hot iron branding. However, Sutherland et al. [15] identified worse physiologic and electroencephalographic response to cryosurgical disbudding as compared to cautery disbudding. Furthermore, Hempstead et al. [3,4,12] found that cryosurgical disbudding in goat kids resulted in elevated serum cortisol, reduced mechanical nociceptive threshold, and an increase in behavioral pain indicators as compared to heat cautery, though less than that observed with caustic paste. There are no known reports of iatrogenic injury secondary to cryosurgical disbudding in goat kids. Cryosurgery can be performed using a liquid nitrogen immersed copper disbudding iron or a commercial cryogen, although it is a relatively slow process. The former technique is inexpensive and utilizes equipment that is readily available and understood by livestock producers in the U.S. The latter technique can be performed with greater precision using a durable cryogenic unit or a relatively new shelf-stable disposable kit. With the emergence of more commercial cryogens on the market, if the technique is consistently safe, effective, and demonstrates rapid healing, freezing may be a feasible approach for goat producers with a net improvement on long-term welfare.

Lastly, a recent advancement in disbudding is the use of clove oil or eugenol injection to prevent horn growth [21,22]. Both studies enrolled small numbers of kids, and did not directly evaluate pain associated with the procedure. However, the authors noted that clove oil and eugenol have anesthetic properties, and subject kids did not appear distressed based on casual observations. Conversely, Frahm et al. [23] reported mechanical nociceptive thresholds increased in kids and calves within 24 h of clove oil or isoeugenol injection. Hempstead et al. [3,12] observed that the acute physiologic and behavioral indicators of pain associated with clove oil disbudding were similar to cautery disbudding, though time to healing was reduced; more concerning, they were not able to fully replicate the efficacy findings of the Molaei et al. study [21]. Hence, although clove oil injection appears to cause less tissue damage than cautery disbudding, there is a critical need to evaluate efficacy and safety of this technique in terms of cumulative impacts on welfare compared to heat cautery disbudding. At this time, clove oil and eugenol are not approved by the FDA for use in food animal species in the United States.

This study comprises one part of a larger project aimed at identifying potential techniques for disbudding kids on commercial dairy goat farms in the absence of anesthesia and analgesia. The specific objectives of the study were to consider the net effect of disbudding technique on goat welfare by examining vocalization frequency, long-term efficacy and animal safety, for three alternative caprine disbudding methods (clove oil injection, one-hour application of caustic paste, or freezing) against sham-disbudded and heat-cautery controls. Assessment of post-surgical nociceptive thresholds, thermography, cortisol and home pen behavior responses are forthcoming.

## 2. Materials and Methods

### 2.1. Animals, Housing and Husbandry

A mixed group of sixty-five healthy, dairy buck kids (predominantly Alpine/cross and Saanen breeds), 1 to 8 days of age, were sourced in three cohorts from a commercial goat dairy in eastern Iowa, USA, and enrolled in the study during February 2017. All kids were provided colostrum at the source farm. On arrival (D-2), the kids were housed together in groups of six in an environmentally controlled room, in pens mounted on plastic raised deck flooring. Each pen represented one replicate of six treatment, with pens blocked for body weight. A 23% protein, 25% fat non-medicated commercial caprine milk (Manna Pro Products, Chesterfield, MO, USA) replacer diet was provided; the milk replacer was reconstituted according to package directions and limit-fed twice daily at 10% of average estimated body weight daily. Hair over the horn buds was clipped, and randomized identification paint markings were applied to the dorsal body. All disbudding treatments occurred two days after arrival (D0). Three days after the disbudding treatment (D+3), kids were transported to a commercial caprine feeder operation, where they were reared in bedded grower barns in group pens of five, maintained on a commercial milk replacer diet, and transitioned to a starter feed at one month of age. The kids were removed from the study at D+45 or at death.

### 2.2. Experimental Treatments

Kids were randomly assigned to one of six disbudding treatments using a random number generator. Information about exact age and breed were not provided from the source farms, and hence treatments were not blocked on these factors. Fewer than six Saanen kids were included in the cohorts, and they were distributed across the treatment groups. Since exact ages were not known for the kids, horn bud volume (mm^3^) was estimated by creating an impression mold with modeling clay on D0 immediately prior to the disbudding treatment; the impression volume was estimated in cc of water and converted to mm^3^. Horn volume did not significantly differ for any of the treatment groups. For all animals, a circular area of approximately 15 mm diameter centered on the horn bud was targeted for treatment.

“HEAT” (n = 11) treated kids were subject to thermal cautery disbudding using a pre-heated 5/8” commercial butane disbudding iron with a sharpened iron tip (Dehorner III, Portasol^®^ USA, Elmira, OR, USA) applied once to each horn bud for no longer than 10 s, followed by complete removal of the horn bud tissue. The manufacturer advertises that this iron reaches a temperature of 1200 F within five minutes of ignition.

“PASTE” (n = 11) treated kids were subject to dry-gauze cleaning of the horn bud, followed by an application of commercial calcium hydroxide 37.8%/sodium hydroxide 24.9% disbudding caustic paste (Dehorning Paste, Dr. Naylor, H.W. Naylor Company Inc., Morris, NY, USA), applied over the horn buds within the confines of a 16 mm petroleum jelly barrier ring. One hour after application, the excess paste was wiped off with dry gauze.

“FREEZE” (n = 11) treated kids were similarly prepared as for CRYOGEN and then a pre-cooled, liquid-nitrogen immersed (minimum of one minute) 5/8” copper disbudding iron was applied once to each horn bud for 40 s, followed by a slow thaw.

“CRYOGEN” (n = 11) treated kids were subject to cleaning of the horn bud with dry gauze, saturation with 99% isopropyl alcohol solution, then the horn buds were shielded with a 16 mm isolation funnel. The horn bud region was flooded with a commercial cryogen compound (Cool Renewal Cyrosurgical Kit, Jorgensen Labs, Loveland, CO, USA) for 10 s, followed by boil off and a slow thaw.

“CLOVE” (n = 11) treated kids were injected with 0.2 mL of >85% eugenol food-grade clove oil (*Eugenia caryophyllata*, food grade certified >85% eugenol 4-ally-2-methoxyphenol, Sigma-Aldrich Corp., St. Louis, MO, USA) injected into the center of each horn bud; the 1” 20-gauge needle was inserted perpendicularly to the skull and rotated during injection to attain a 360-degree injection distribution.

“SHAM” (n = 10) disbudding control kids were subject to cleaning of the horn bud with dry gauze, saturation with 99% isopropyl alcohol solution, and firm pressure application of a room-temperature ½” commercial disbudding iron for ten seconds to each horn bud.

Treatments were performed by the same veterinarian, on the same day per cohort group, administered in random order within the group pen, and performed with the kids individually restrained in a disbudding box. With the exception of “PASTE” treatments, all kids were immediately returned to their pen group. PASTE treated kids were housed individually for one hour, after which paste was removed and the kid was returned to the home pen. The subject and pen cohort were observed daily through D+3 for evidence of paste transfer injury.

### 2.3. Data Collection

Vocalizations: Vocalization was used as a readily observable indicator of distress during the disbudding procedure. Vocalization score refers to the total number of vocalizations during the disbudding procedure for both horn buds. Frequency of unique spontaneous vocalizations was recorded by the same observer during the time of the procedure for each goat. Vocalizations were counted continuously from the moment the veterinarian made first contact with the first horn bud and ended when final contact with the second horn bud ceased.

Horn Bud Tissue Scoring: The same observer visually scored the horn bud tissues daily for D−1 to D+3 and then weekly for D+10 to D+45. The horn buds were scored for inflammation, necrotic escher, purulent discharge, scab, or horn/scur growth at each observation point. On removal from study (D+45 or at death) horn or scur maximal width (mm) and height (mm) were measured with calipers as an end point for horn/scur growth and volume estimated as a simple product of the two. Disbudding success was characterized by a complete absence of horn growth from the primary horn bud at forty-five days post disbudding treatment; disbudding failure was characterized by presence of a primary horn bud scur or normal horn growth. Small scurs along the medial ridge were included in terminal scur volume assessment, but were not considered disbudding failures. Scurs were defined as any horn growth from the primary horn bud, and included observations of normally formed horns.

In the U.S., clove oil is considered an unapproved animal drug and as such, is not permitted for injection into a food animal species. For this reason, all CLOVE subjects were euthanized at the conclusion of the study on D+45. CLOVE subjects and all spontaneous fatalities were submitted for a routine complete gross necropsy at the Iowa State University Veterinary Diagnostic Laboratory.

### 2.4. Statistical Analysis

The experimental unit was the kid. Based on a priori sample size calculations, ten kids per treatment were needed to provide sufficient statistical power to reject the null hypothesis with 25% difference in efficacy relative to HEAT, β = 0.8, and α = 0.05. In all analyses, α = 0.05 was the threshold for significance, whereas 0.05 < α < 0.10 was considered a tendency or trend.

Vocalization data were log transformed and subject to a one-way ANOVA and Dunnett’s test (familywise α = 0.05) using open-source statistical software; the analysis included fixed effect of treatment group without additional covariates.

A one-tailed Fisher’s Exact test was used to compare frequency of disbudding successes and complications, with each horn representing an observation. A one-tailed Student’s T distribution was used to evaluate the total resulting horn or scur, with each kid representing an observation.

### 2.5. Ethical Note

The Iowa State University Institutional Animal Care and Use Committee (1-17-8416C) provided protocol approval and study oversight in accordance with U.S. Federal Animal Welfare Regulations (CFR Ch 1, 2.36(b) 5–7).

The decision to not provide pain relief through anesthesia or analgesia was given serious consideration, and was based on balancing external validity of our findings for immediate application to existing agricultural practices in North America. Furthermore, including additional and desire to reduce the number of treatment groups and animals required to answer our research question.

We also note that five subjects died unexpectedly between D+35 and the study termination on D+45. These adverse events were not anticipated based on scientific reports of these disbudding techniques, and were reported to the IACUC. Post mortem analyses were performed for all mortalities.

## 3. Results

### 3.1. Vocalizations

**Figure 1:**
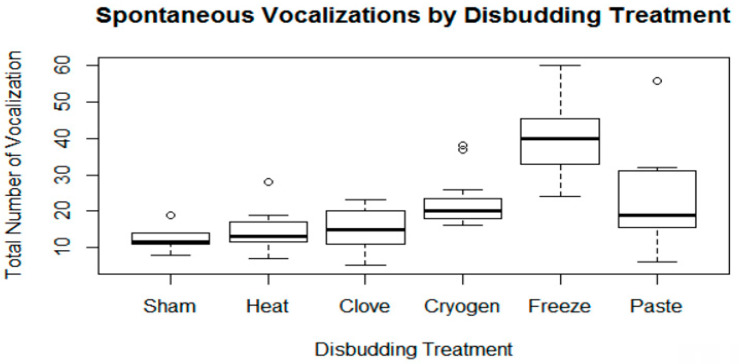
Total vocalizations performed during disbudding treatment. Boxes represent quartiles and median values; whiskers are min/max up to 1.5× the interquartile range; open circles represent outliers.

There was an effect of treatment on vocalization score (Figure 1). Vocalizations were 2.8, 3.0 and 7.3 times higher during CRYOGEN (*p* = 0.0315), FREEZE (*p* < 0.001) and PASTE (*p* = 0.0196) treatments relative to SHAM. There were no differences between SHAM and HEAT or SHAM and CLOVE treatments. When compared to HEAT, vocalizations were 6.8 times higher during FREEZE (*p* < 0.001). Vocalizations did not differ between HEAT, CLOVE, CYRO, and PASTE. FREEZE differed from all other treatments.

### 3.2. Efficacy

Efficacy of treatment is summarized in Table 1. HEAT was more effective than PASTE (91% and 54.5% success respectively, *p* < 0.05), and efficacy of HEAT and PASTE were each superior to SHAM CLOVE, FREEZE and CRYO (*p* < 0.0001). Disbudding failure with HEAT or PASTE was associated with larger horn, and efficacy of PASTE was reduced when cornified tissue was present at the time of disbudding. CLOVE resulted in 91% scurs and 9% normal horns. Although CLOVE was not more effective than SHAM in preventing any growth of horned tissue, it did result in 47% less horn volume at 45 days post-treatment (*p* < 0.005). The scurs associated with CLOVE were uniquely formed, with a large central cavitation and relatively normal peripheral horn production. HEAT resulted in fewer scurs (9%) than PASTE (27%) or CLOVE (*p* < 0.05).

**Figure 2:**
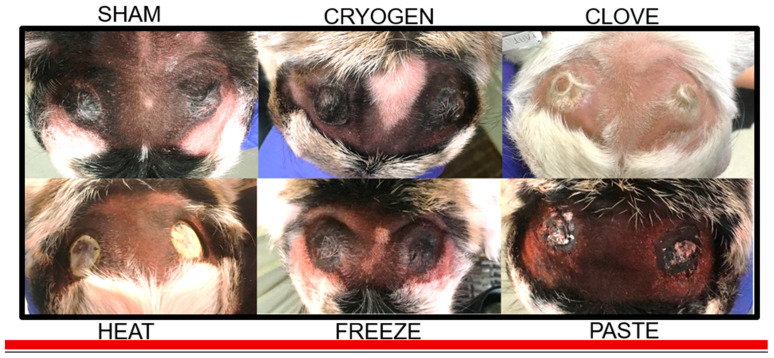
Representative images of the horn bud tissues from each treatment group on D+2. Normal closed dry horn bud tissue is observed in the SHAM image. Open cauterized margins of the excised horn bud are observed in the HEAT image. A closed dry ring of necrotic tissue can be observed at the base of the horn bud in the CRYOGEN and FREEZE images with additional erythema indicative of inflammation in the latter; open wet necrotic tissue over the central horn bud and edema/erythema indicative of inflammation can be observed in the PASTE image. Closed dry soft tissue swelling and discoloration indicative of underlying tissue necrosis can be observed around the base of the horn bud in the CLOVE image.

**Figure 3:**
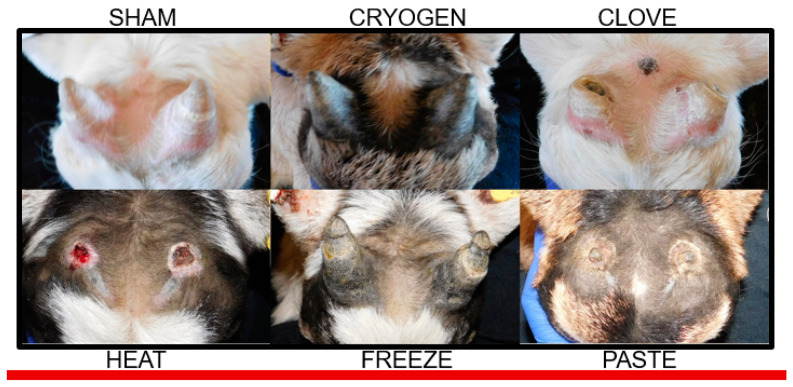
Representative images of the horn bud tissues from each treatment group on D+25. Normal horn growth is observed in the SHAM image along with failure of the disbudding procedure in the CRYOGEN, FREEZE, and CLOVE treatments. The slight concentric deformity of the horn tip appreciated in the CRYOGEN and FREEZE images is most likely restricted to insensitive tissues. Large abnormal scurs with central cavitation can be observed in the CLOVE image, in some cases these central necrotic cavitations extended through the skull and created conditions for dural abscesses or meningoencephalitis. The superficial loose tissue necrosis with underlying healing epidermis observed in the PASTE image is indicative of a successful and nearly healed procedure. A normal healing open epidermal lesion with incomplete re-epithelialization and scalp can be observed in the HEAT image, these lesions are expected to have a reduced mechanical nociceptive threshold until re-epithelialization is complete.

Effects of disbudding treatment on horn bud tissues are described in Table 2, including appearance of the horn bud tissues at D+3 and D+10, the time of first observed complete re-epithelialization after scab or eschar formation, the frequency and time distributions of first reported purulent discharge, and the observed outcomes at D+45. Effects of treatment on tissue healing on D+2 and D+25 are illustrated in Figure 2 and Figure 3, respectively.

### 3.3. Safety

Complications of treatment are summarized in Table 3. HEAT resulted in more superficial infections at the disbudding site (64%, *p* < 0.005) than SHAM, characterized primarily as purulent discharged under the horn bud scab and did not result in any serious complications. These were effectively treated by one-time superficial debridement of the scab and purulent material with an antiseptic-soaked gauze. There were no clinically observed signs of thermal encephalitis in the HEAT treated kids. There were no differences in disbudding-related complications between CRYO, FREEZE, and SHAM. There was no evidence of paste transfer injury among the PASTE treated kids or their pen cohorts.

**Figure 4:**
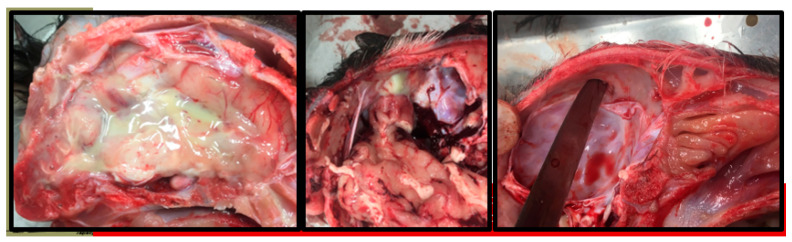
Suppurative meningitis, dural abscess, and calvarium defect observed following the CLOVE treatment. These were observed both in spontaneous mortalities (e.g., suppurative meningitis) and in grossly healthy kids euthanized at the study conclusion (e.g., dural abscess, calvarium defect).

One CLOVE treated kid demonstrated severe orbital swelling on D+1, which progressed to necrosis of the upper eyelid margin. Three CLOVE kids demonstrated transient flaccid tetraparesis and paralysis immediately after injection; mentation was not affected and the condition spontaneously self-resolved within 10 min of the clove oil injection. All three kids exhibiting tetraparesis had more advanced horn bud development (>0.2 cc horn bud volume) on D0 as compared to the other CLOVE treated kids. Fatal complications occurred in four out of the eleven CLOVE animals, more than other treatment groups (*p* = 0.045). Evidence of suppurative meningitis, dural abscess, and/or complete defect in the calvarium was observed post-mortem in three additional CLOVE kids (Figure 4). Two deaths in the PASTE and FREEZE treatment groups were attributed to enterotoxemia and chronic failure to thrive, respectively, and were not related to treatment. All deaths across treatments occurred after D+35.

## 4. Discussion

The vocalization comparisons indicate that FREEZE, CRYO, and PASTE may create a greater total effect of acute distress than HEAT or SHAM controls at the time of the disbudding procedure; further investigation into behavioral and physiologic response during the disbudding process is indicated. No difference was observed with CLOVE as compared to HEAT or SHAM. Surprisingly, there was also no difference between HEAT and SHAM, suggesting that the total vocalization count may reflect distress associated with restraint and duration of the procedure rather than or in addition to pain. Whereas pain associated with heat cautery is well established, it is a relatively quick procedure to perform. In comparison, the cryosurgical methods required prolonged application of the cold iron. An alternate explanation is that the pain associated with heat cautery produced few, longer vocalizations and paradoxically depressed the total vocalization count; in the author’s estimation this is less likely. While vocalization is a readily observed distress indicator that is likely to influence caretaker protocols, it is not a good direct indicator of pain. Lay et al. [16,17] did not find significant differences in vocalization scores during hot and cold branding; escape-avoidance reactions were a more sensitive indicator of distress and should be further investigated for disbudding. However, based on vocalization frequency alone there is no evidence that alternate methods are superior to heat cautery and FREEZE was significantly worse. Our research team is conducting further examination of post-surgical pain responses, including mechanical nociceptive thresholds, infrared thermography, home pen behavior and cortisol, which will complement the current work.

Heat cautery was the most efficacious method and least affected by horn bud volume at the time of disbudding. The most common associated scur growth was along the medial horn ridge, which is a location that is notoriously difficult to effectively disbud in male dairy goats. Producers often perform a secondary burn in a figure-eight style pattern to prevent those scurs in males [6] or utilize tear-shaped buck tips; in order to maintain consistency between treatment groups, those methods were not employed in this study. The relatively high number of superficial infections observed in this study compared to that reported by Hempstead et al. [7] supports research to identify and mitigate risk factors related to equipment, technique, adjunct therapy, and environment. For instance, disbudding irons are typically divided into sharp, cutting tips where the central horn bud tissue is fully removed and broader tips, where cautery occurs over the surface of the horn bud without cutting the skin. Although the method used by Hempstead et al. [7] and this study were similar in use of the narrow cutting tips, to the authors knowledge there is no information directly comparing the major approaches to cautery disbudding with respect to wound infection. It is possible that the cutting tips create a higher risk of infection, especially if the skin margin is incompletely cauterized. Furthermore, some practitioners routinely use topical bandages or salves of unknown efficacy on the open disbudding wounds; these were not applied in this study in order to avoid masking observation of the horn bud tissue. In all cases, the superficial infections were resolved by the next weekly observation after removal of the overlying scab and cleaning with mild topical disinfectant solution. It is unclear to what degree these infections directly increase pain; the area appeared moderately sensitized to touch at the time of presentation and any wound infection is expected to increase time to complete healing and re-epithelialization. Methods that improve welfare and reduce risk of iatrogenic injury are desired. However, the heat cautery procedure had the advantage of being both quick and effective as compared to the alternatives tested.

Short-term application of caustic paste was effective, but significantly more prone to failure than heat cautery in presence of cornified tissue. Application of caustic paste for a shorter, defined period, allowed for isolation of the disbudded animal from peers; the absence of paste transfer once returned to group housing suggests that this technique may be safely adapted to group housed and dam-raised animals. Efficacy of caustic paste disbudding in kids is not well described in the literature; Hempstead et al. [4] described open, wet wounds, with eschar formation and scab persistence through to six weeks and scur formation in six of ten kids. Our study found a similar rate of efficacy and duration of wound healing but more moderate observations on wound size and severity. Although the sample size was limited in both of these studies, the results suggest that the one-hour application was effective, and did reduced risk of paste transfer injuries, but that the caustic paste method of disbudding has an unacceptably high risk of failure in goats. In contrast, in calves Newby et al. [24] found that efficacy of traditional application of caustic paste disbudding did not differ from heat cautery, with no scur formation after six weeks in 96 calves. One reason for the species difference in outcomes may be the relative rate at which goats and cattle produce cornified horn; it is possible that further investigation in very young animals, prior to any cornification of the horn bud, would yield different results. However, we can conclude that this procedure is not benign from a welfare standpoint. Compared to heat cautery, caustic paste application took longer to perform, required a period of social isolation, and casual observation identified behavioral indicators of discomfort that are consistent with other reported behavioral, physiological, and nociceptive observations of pain following this procedure [3,4,12].

Based on the high frequency of scur formation and unacceptably severe complications observed in this study, serious concerns are raised about the use of clove oil injection for caprine disbudding. Unfortunately, the results of 100% success with clove oil disbudding reported by Molaei et al. [21] could not be replicated. Similar work presented by Hempstead et al. [7] reported a probability of success for clove oil disbudding of 9%. One plausible rational is that the differences in observed efficacy are related to breed-related rates of horn growth in the subject populations; certainly, continental-breed dairy male kids are known to grow horn rapidly from a very early age and as a result are typically disbudded in the first week or two of life. Reports on short-term efficacy of clove oil disbudding in calves [14,25] are much more favorable, lending credence to this theory, though recent work examining long-term efficacy of clove-oil injection in cattle ultimately demonstrated delay rather than prevention in scur/horn growth [26]. A second rational is that the source of clove oil may have differed between the studies; however, we did source a tested and certified product containing at least 85% eugenol that is consistent with both Molaei et al.’s approach and the work performed by Hempstead et al. and the volume injected was similar between the three studies.

Several serious and unexpected complications were observed following clove oil injection in this study. Although these complications were not reported by Molaei et al. [21], Hempstead et al. [7] also reported acute periorbital swelling following clove oil injection into the horn bud in several kids. However, subsequent tissue necrosis beyond the horn bud was not observed in their study. They did not attempt to further characterize if the observed swelling also increase tissue sensitivity and pain perception. With only the one observation in our study the overriding concern is the effect of irreversible eyelid margin necrosis on long-term ocular health and animal welfare. The finding of transient flaccid tetraparesis has not previously been reported in goat kids, but is consistent with its role as a known dose-dependent anesthetic in several invertebrate and vertebrate species; eugenol also has demonstrated cardiovascular depressive and hypotensive effects [27]. Since this observation was limited to kids with more advance horn development, and the vascular bed underlying the horn bud is more robust once the horn starts emerging, it is possible that a portion of the clove oil injection in those subjects entered vascular circulation. If so, the risk of tetraparesis may be minimized by limiting clove oil injection to perinatal kids prior to emergence of the horn.

The most serious observations associated with clove oil injection included defects in the calvarium under the horn bud, dural abscesses, and diffuse suppurative meningitis. While Hempstead et al. [7] did not report similar complications, at least two Nigerian Dwarf kids treated with clove oil in practice have died from similar skull necrosis following injection (private communication). It is unclear if this observation is related to compound, dose, or injection method. However, given the similarities in compound and dose between our study, Hempstead et al. [7] and Molaei et al. [21], the most likely explanation is in injection technique. We used a needle placement perpendicular to the plane of the skull and rotated the needle bevel to distribute the clove oil; depth of needle placement was confirmed by feel and it is conceivable that the needle was advanced during injection, resulting in periosteal injection. In contrast, Hempstead et al. [7] used a needle placement parallel to the plane of the skull, which presented separate challenges, but less risk of increased depth of injection. Molaei et al. did not specify their needle placement approach. If so, this suggests that tissue plane of injection is critical and minor differences in injection technique can produce a wide range of potentially fatal complications. Further research is needed to identify potential associations with technique and/or study population before clove-oil injection can be recommended. However, it is important to note that in addition to the established safety and efficacy concerns, clove oil cannot be legally injected into food-producing animals in the United States; although generally recognized as safe for use in food, in GFI #150 the FDA Center for Veterinary Medicine has published clear guidance to industry against the use of clove oil as an animal drug [28].

As performed, both methods of freezing were ineffective. While it is possible that the technique employed was insufficient to kill the germinal epithelium, previous trials on contemporary cadaver skulls created horn bud tissue temperatures below fifty degrees centigrade (unpublished data). While inclusion of longer freezing intervals or repeated freeze-thaw cycles may improve efficacy, especially in very young neonates, the extended time is likely to increase procedural stress for the animal and prove impractical in a production setting. Given that Hempstead et al. [3,4,12] found poorer outcomes for physiological markers of distress, increased pain sensitivity, and increased behavioral pain indicators in goats disbudded with cryosurgery, and that we observed the highest vocalization frequency and lowest treatment efficacy in these techniques, it does not appear that cryosurgical methods are likely to improve welfare outcomes compared to heat cautery disbudding.

## 5. Conclusions

This study comprises one part of a larger project aimed at identifying potential techniques for disbudding kids on commercial dairy goat farms in the absence of anesthesia and analgesia; here we focused on efficacy and complications of disbudding techniques. Of the disbudding treatments tested, heat cautery disbudding demonstrated the greatest efficacy of disbudding success, and did not result in serious or irreversible complications in this study. In comparison, the protocol of short-term application of caustic paste used in this study was only marginally effective; employing this method would result in an unacceptable requirement for secondary dehorning procedures. Other caustic paste protocols exist that may be more effective for both disbudding and preventing paste transfer injury. Similarly, cryosurgical disbudding methods were ineffective and were associated with high numbers of vocalizations. Clove oil injection cannot be recommended as it created large, abnormal horn scurs, presented a significant risk of serious or fatal complications, and is not legal for use in the United States. On the basis of poor efficacy and risk of complications, we were unable to identify an alternative disbudding technique suitable for on farm use in the absence of analgesia.

## Figures and Tables

**Table 1:** Disbudding efficacy. For each of the six treatment groups, the mean baseline horn bud volume prior to disbudding, the frequency of successful disbudding treatments, and the mean volume (height × width) of any horn or scur growth at study termination are reported below.

Treatment Group	D-1Horn Bud Volume, cc Mean (SE)	% Success at D+45 ((n) Success/Failure)	Scur Volume at D+45, mm^3^ Mean (SE)
SHAM (n = 10)	0.31 (0.08)	0% (0/20)	2327 (491)
HEAT (n = 11)	0.28 (0.07)	91% (20/2 ^a,b^)	9 (8)
PASTE (n = 11) ^%^	0.20 (0.05)	55% (12/10 ^a^)	1902 (521)
CLOVE (n = 11)	0.26 (0.08)	0% (0/22)	2328 (491)
FREEZE (n = 11) ^%^	0.28 (0.08)	0% (0/22)	4413 (748)
CRYOGEN (n = 11) *	0.31 (0.09)	0% (0/22)	3494 (395)

^a^ Differs from SHAM, *p* < 0.0001. ^b^ Differs from PASTE, *p* = 0.0157. * n = 4 kids removed from the study for complications prior to D+45. ^%^ n = 1 kid died prior to D+45.

**Table 2:** Raw data describing effects of disbudding treatment (D0) on horn bud tissue. Observations are reported at the horn bud level; mortalities prior to D+45 are censored.

Treatment	D+3	D+10	First Observed Complete Re-epithelialization	First Report of Purulent Discharge	At D+45
SHAM (n = 20)	Normal Epidermis (Closed and Dry)	Normal Horn	n/a	n/a	Normal Horn (n = 20)
HEAT (n = 22)	Open Dry	Scab Dry to Purulent	D+24 (n = 1) D+31 (n = 4) D+38 (n = 4) D+45 (n = 12)	D+10 (n = 4) D+17 (n = 1) D+24 (n = 1) D+31 (n = 1) D+38 (n = 1) D+45 (n = 1)	Healed (n = 19) Purulent (n = 1) Scur Horn (n = 2)
PASTE (n = 22) ^%^	Open & Necrotic Wet	Normal, Necrotic, or Eschar Dry Scur Horn (n = 6)	D+38 (n = 3) D+45 (n = 6)	D+17 (n = 1) D+38 (n = 1)	Healed (n = 9) Scab (n = 1) Normal Horn (n = 4) Scur Horn (n = 6)
CLOVE (n = 22) *	Closed & Necrotic Dry	Normal to Necrotic Dry Scur Horn (n = 8)	D+10 (n = 2) D+17 (n = 2) D+38 (n = 1)	D+17 (n = 1) D+31 (n = 2) D+38 (n = 1)	Normal Horn (n = 2) Scur Horn (n = 12)
FREEZE (n = 22) ^%^	Closed, Normal to Necrotic Occasionally Wet	Normal to Necrotic Dry Scur Horn (n = 20)	D+24 (n = 2)	n/a	Normal Horn (n = 20) Scur Horn (n = 2)
CRYOGEN (n = 22)	Closed, Normal to Necrotic Dry	Normal to Eschar Dry Scur Horn (n = 18)	D+10 (n = 1) D+17 (n = 1)	D+31 (n = 1)	Normal Horn (n = 13) Scur Horn (n = 9)

* n = 4 kids removed from the study for complications prior to D+45. ^%^ n = 1 kid died prior to D+45.

**Table 3:** Disbudding safety. For each of the six treatment groups, the frequency of superficial horn bud infections, the mortality rate prior to study termination, and the frequency of post-mortem disbudding related lesions are reported below.

Treatment Group	% (n) Superficial Infection	% (n) Died Prior to D+45	% (n) Post-Mortem Abnormalities
SHAM (n = 10)	0% (0)	0% (0)	n/a
HEAT (n = 11)	64% (7) ^a^	0% (0)	n/a
PASTE (n = 11) ^%^	9% (1)	9% (1)	0% (0)
CLOVE (n = 11)	27% (3) ^b^	36% (4) ^c^	64% (7)
FREEZE (n = 11) ^%^	0% (0)	9% (1)	0% (0)
CRYOGEN (n = 11) *	9% (1)	0% (0)	n/a

^a^ Differs from SHAM (*p* = 0.0028). ^b^ Tendency to differ from HEAT (*p* = 0.0992). ^c^ Differs from HEAT/FREEZE, and CRYOGEN (*p* = 0.0451) and tendency to differ from SHAM (*p* = 0.0551). * n = 4 kids removed from the study for complications prior to D+45. ^%^ n = 1 kid died prior to D+45.

## Data Availability

Data available upon request due to restrictions eg privacy or ethical.
